# Introduction and acceptability of the Surveillance Outbreak Response Management and Analysis System (SORMAS) during the COVID-19 pandemic in Côte d’Ivoire

**DOI:** 10.1186/s12889-023-17026-3

**Published:** 2023-11-07

**Authors:** Tanja Barth-Jaeggi, Clarisse A. Houngbedji, Marta S. Palmeirim, Daouda Coulibaly, Aboubakar Krouman, Cordula Ressing, Kaspar Wyss

**Affiliations:** 1 Swiss Tropical and Public Health Institute (Swiss TPH), Allschwil, Switzerland; 2 University of Basel, Basel, Switzerland; 3 Centre d’Entomologie Médicale Et Véterinaire (CEMV), Université Alassane Ouattara, Bouaké, Côte d’Ivoire; 4 Centre Suisse de Recherches Scientifiques en Côte d’Ivoire (CSRS), Abidjan, Côte d’Ivoire; 5 Institut National d’Hygiène Publique (INHP), Abidjan, Côte d’Ivoire; 6 Helmholtz Centre for Infection Research (HZI), Brunswick, Germany

**Keywords:** COVID-19, Outbreak surveillance, Outbreak management, Health informatics, Software, SORMAS, Côte d’Ivoire

## Abstract

**Background:**

The Surveillance Outbreak Response Management and Analysis System (SORMAS) has been implemented for various infectious diseases since 2015. 2020, at the beginning of the COVID-19 pandemic, SORMAS was adapted to SARS-CoV2.

**Methods:**

We assessed the acceptability and usability of SORMAS and accompanied its implementation in two pilot regions of Côte d’Ivoire (Abidjan 2 and Gbêkê) from July/August 2021 to March 2022. We conducted 136 semi-structured interviews to cover knowledge on COVID-19, information on conventional surveillance systems for disease monitoring including COVID-19, acceptability of SORMAS, and impact of SORMAS on epidemic preparedness and surveillance. Scores before and 6–8 months after implementation were compared.

**Results:**

SORMAS was implemented in two pilot regions in Côte d’Ivoire. The conventional software for the surveillance of the COVID-19 pandemic by the company MAGPI was maintained in parallel; the additional time needs to enter and manage the data in SORMAS were the main concern. SORMAS acceptance and satisfaction scores were high after the user training, which was prior to implementation, and after 6–8 months of use. The ability of SORMAS to improve COVID-19 preparedness and early detection of cases and contacts was widely acknowledged. To keep the understanding and skills of users up-to-date, regular refresher trainings were requested. The expectation to be able to make decisions based on data produced by SORMAS was high at baseline and the perceived experience after several months of use of the software was very positive. Unfortunately, the link with the laboratories could not be established in the pilot regions, but it is an existing feature of SORMAS that many users were asking for. Following the positive experience using SORMAS for COVID-19, the pilot regions expanded its use for monitoring and management of measles, yellow fever, meningitis, and cholera.

**Conclusion:**

SORMAS was very well accepted by users and decision makers in the two pilot regions of Côte d’Ivoire and its ability to improve epidemic preparedness and surveillance was acknowledged. If the hurdles of maintenance (tablets, server, and maintaining user skills) are handled sustainably, it can serve as a valid tool to identify, surveil and manage future outbreaks of various infectious diseases in Côte d’Ivoire.

## Background

**Fig. 1:**
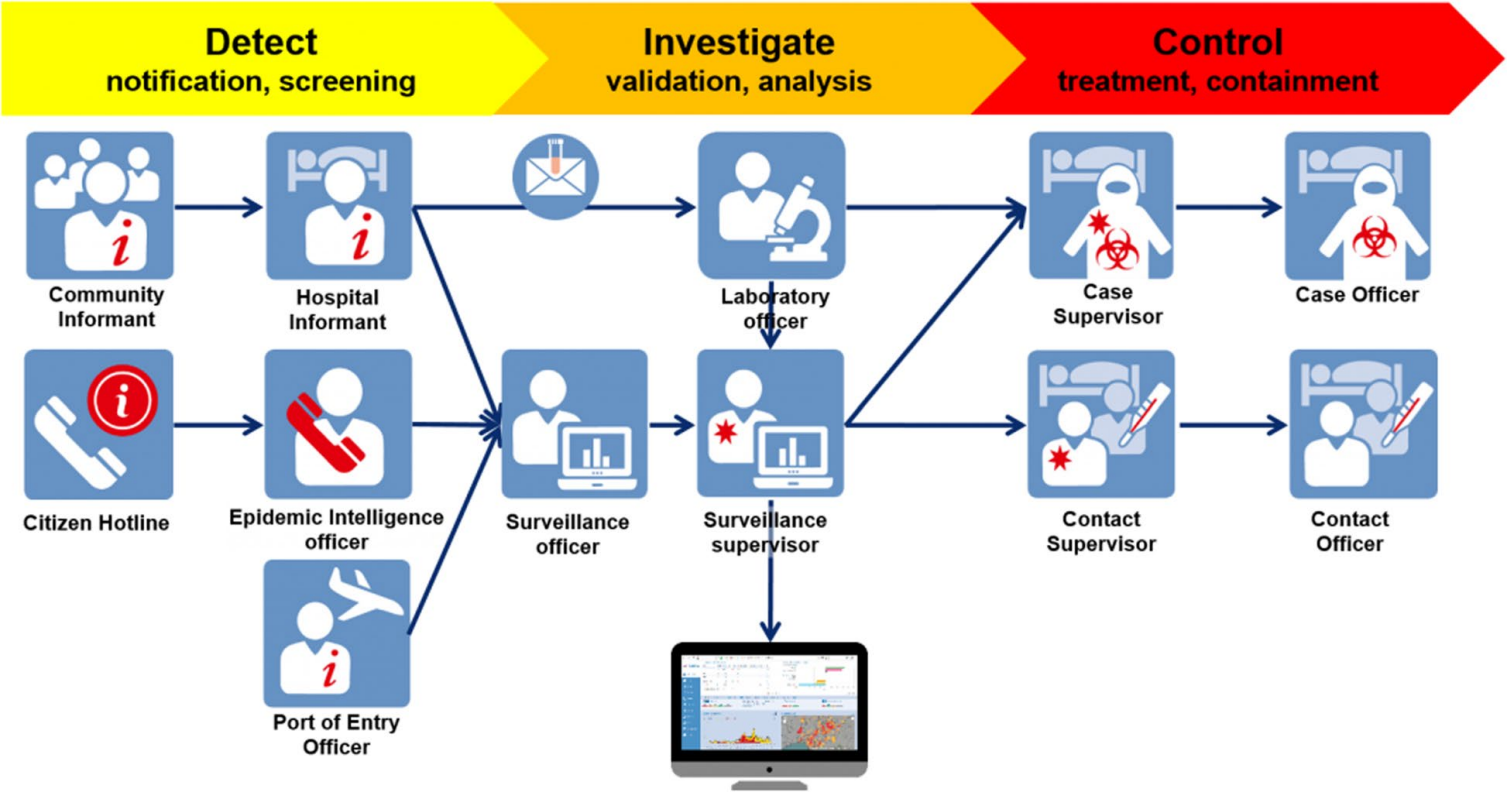
SORMAS software allows simultaneous workflow and interlinkages. Illustration by the Helmholtz Center for Infection Research

The software SORMAS (Surveillance Outbreak Response Management and Analysis System) was developed by Nigerian and German partners as part of their experience during the Ebola virus outbreak in West Africa in 2014–2015. SORMAS is an open source mobile and web application that enables healthcare workers and surveillance officers to notify health services or politicians and decision makers of new cases of infectious diseases, and to manage the response to such outbreaks or epidemics. SORMAS covers 43 diseases and provides disease-specific process models (with algorithms for case definitions and classifications, implemented in line with WHO standard guidelines) for 16 of them. Furthermore, it offers specific interfaces for 12 different types of users, such as clinicians, epidemiologists and laboratory workers (see Fig. 1). SORMAS is free of cost and respects the highest data protection standards, good scientific practice and the open access policy. SORMAS’ vision is to improve the prevention and control of communicable diseases, especially in low-resource settings, and should be customized in collaboration with those involved in public health surveillance and monitoring disease control.

In 2017, soon after its deployment in Nigeria, SORMAS successfully contributed to the response to three simultaneous large outbreaks caused by monkey pox, Lassa fever and bacterial meningitis. Publications in high impact peer-reviewed journals highlight the usefulness of SORMAS for preparedness and response in Nigeria [1–3]. Since then, the number of clinicians, nurses, laboratory technicians, public health workers, and epidemiologists using SORMAS on mobile tablets or desktop computers has continued to grow. Currently, SORMAS is used by healthcare professionals on a routine national or subnational level in Germany, Ghana, and Nigeria. In Luxemburg, SORMAS is currently being implementing at national level for all notifiable diseases. On a subnational level, it was used during the COVID-19 pandemic in Cameroon, Central African Republic, France, Fiji, Gabon, the Republic of the Congo, the Republic of Chad, and Switzerland. Additionally, it is in pilot phase in Côte d’Ivoire, Nepal, Tanzania, and Tunisia.

A study by Tom-Aba and colleagues published in 2018 compared SORMAS to other similar tools in terms of functionality and technical characteristics, and assessed user perception, acceptance and use during the Ebola disease outbreak in West Africa 2014–2015 [4–6]. Healthcare professionals have found SORMAS to be very useful, acceptable and have reported improvements over time.

**Fig. 2:**
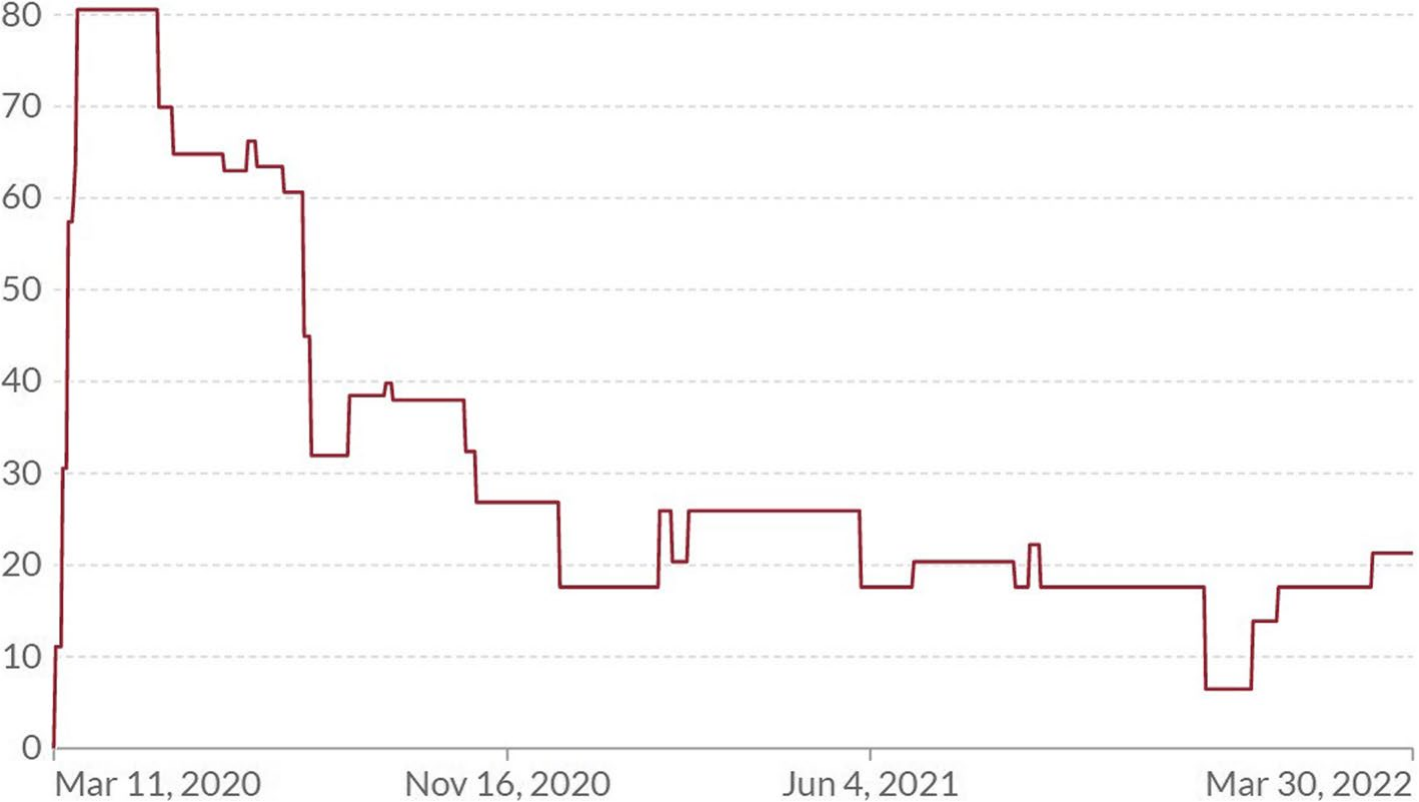
COVID-19 Stringency Index Côte d’Ivoire [10]

On 11 March 2020, the World Health Organization declared the COVID-19 outbreak a pandemic. In Côte d’Ivoire, the first case of COVID-19 was diagnosed on 21 March 2020 [7]. Under the lead of the “Institut National d’Hygiène Publique”, contact tracing was immediately established from this point onwards. On 23 March 2020, the Government of Côte d’Ivoire declared the countrywide state of emergency and reported 73 cases, which rapidly increased to 916 cases and 13 deaths by 21 April 2020. At this point, the country’s borders were closed and all movement of people to and from Abidjan was banned, along with the introductions of a national curfew (9pm to 5am) and closure of all bars and schools [8]. On 16 September 2020, Abidjan ended isolation and schools reopened. Furthermore, the country opened the air borders for travelers with a negative COVID-19 test certificate. The wearing of masks in public places remained in place [9]. The Oxford Stringency Index for Côte d’Ivoire, a composite measure based on nine response indicators including school closures, workplace closures, and travel bans, rescale to a value from 0 to 100 (100 being the strictest), over time is shown in Fig. 2 [10].

**Fig. 3:**
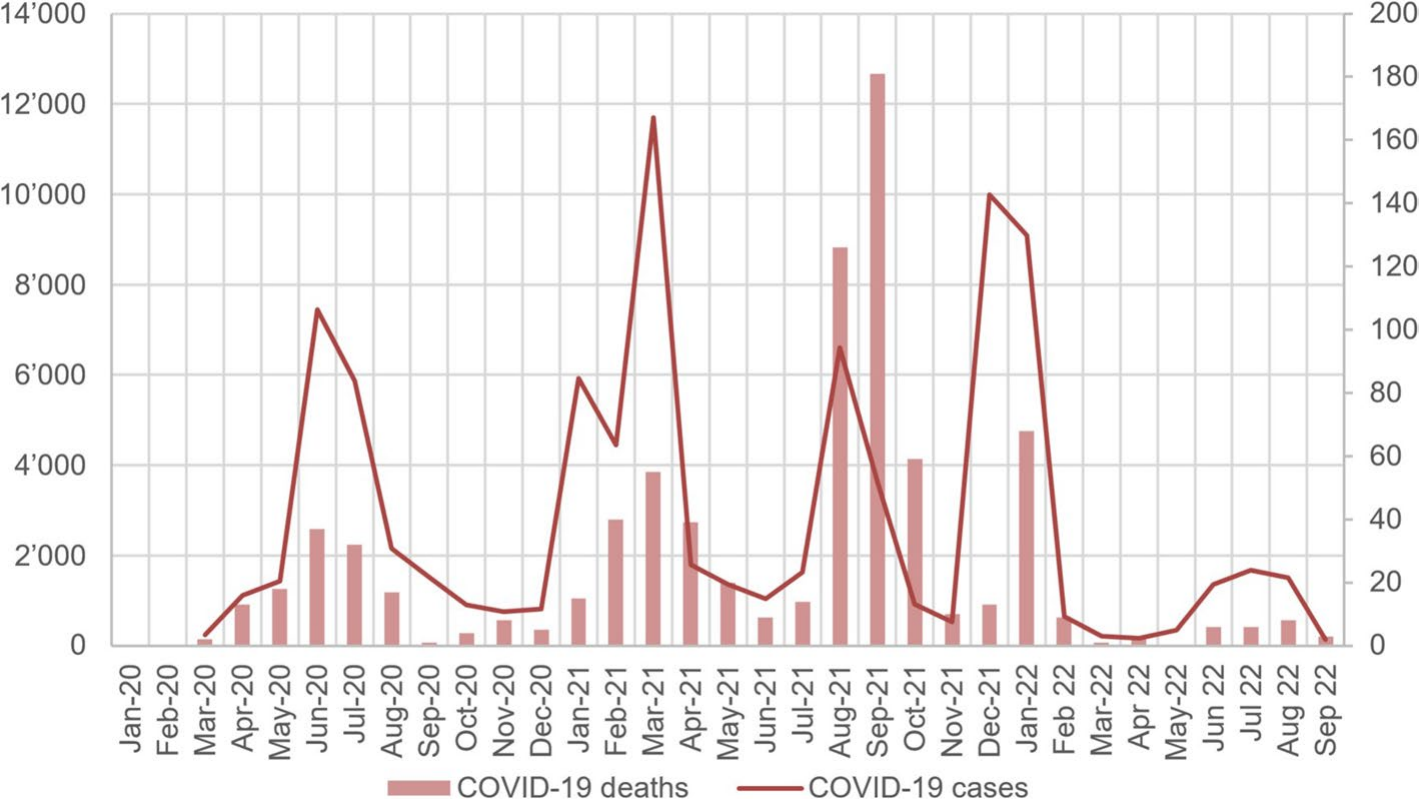
COVID-19 cases and deaths in Côte d’Ivoire [11]

By the end of March 2022, the country reported 82,280 diagnosed cases and 799 deaths [11]. Since the beginning of 2022, the cases of COVID-19 recorded in Côte d’Ivoire have dropped considerably (Fig. 3) [11]. The COVID-19 pandemic put immense pressure on health systems worldwide, with the rapid increase in demand for health services and workers' health [12]. Today, there are several vaccines to protect against it. As of end of March 2022, in Côte d’Ivoire, 16.9% of the population (4.6 million) completed the initial vaccination protocol (https://github.com/owid/covid-19-data/tree/master/public/data/vaccinations).

Throughout the pandemic, the surveillance strategy of Côte d’Ivoire is based on screening for SARS-CoV-2 by rt-PCR (real-time polymerase chain reaction); testing of symptomatic persons, contacts of confirmed cases, persons with comorbidities and travelers. In addition, rapid antigenic tests have been made available to hospitals to enable timely management of patients. According to national guidelines, positive samples should be confirmed by PCR. For the surveillance and management of the COVID-10 pandemic, Côte d’Ivoire used either a surveillance software by the company MAGPI or District Health Information System 2 (DHIS2). Both of these tools were used at national-scale for the surveillance of several diseases. But they do have their limitations, both of them allow only limited data analysis, MAGPI does not allow contact tracing and DHIS2 does not have the link to the laboratories to receive test results.

Therefore, in the frame of the European Union funded CORESMA project (COVID-19 Outbreak Response combining E-health, Serolomics, Modelling, Artificial Intelligence and Implementation Research), SORMAS was implemented for COVID-19 surveillance in two pilot areas in Côte d’Ivoire. This study’s objective is to accompany the implementation and assess the acceptability and usability of SORMAS by users and COVID-19 decision makers in two pilot regions of the Côte d’Ivoire.

## Methods

### Study area and participants

**Fig. 4:**
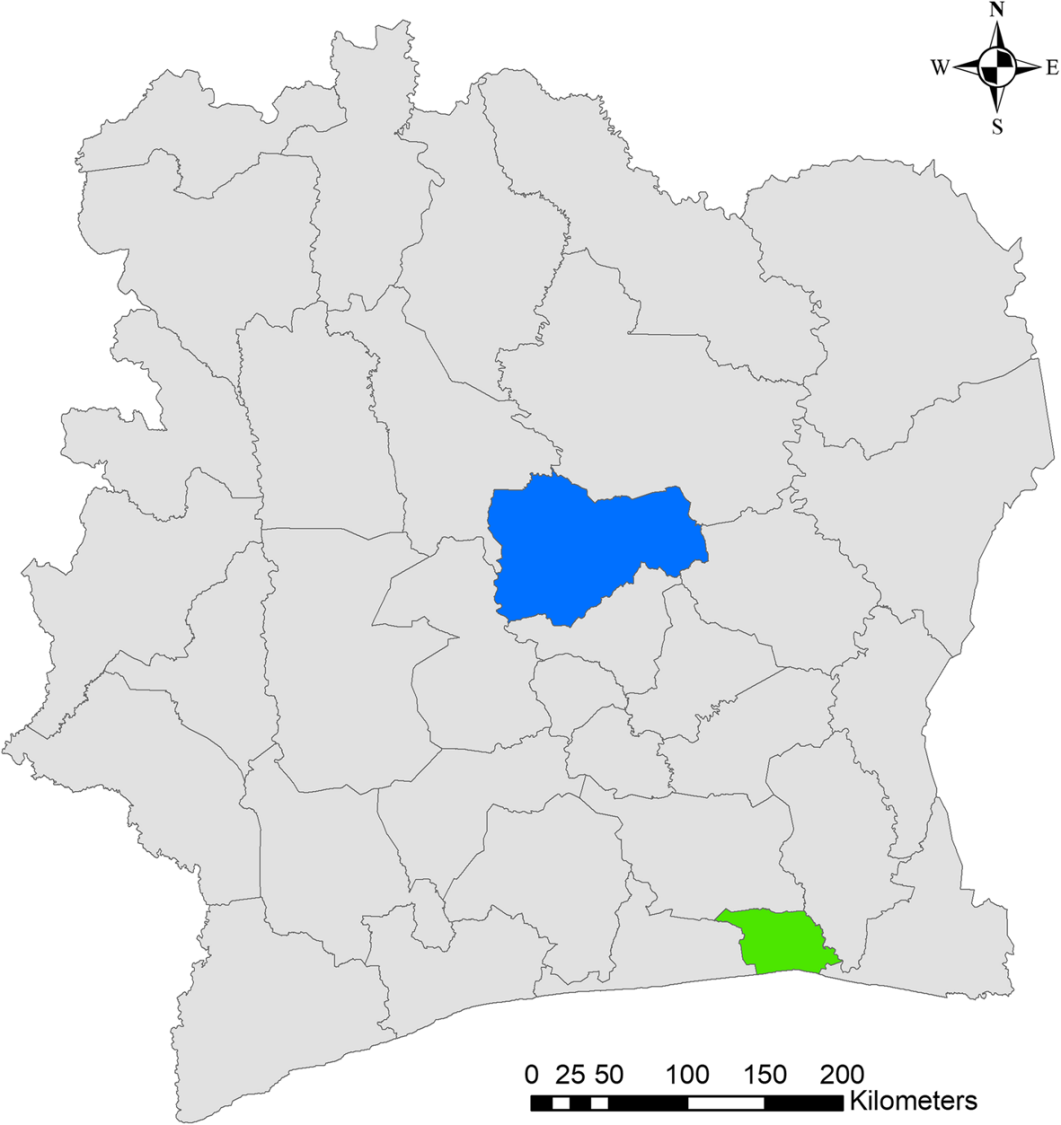
Map of study regions Abidjan (green) and Gbêkê (blue)

For the pilot implementation of SORMAS in Côte d’Ivoire, two health regions were selected: urban Abidjan 2 in the South with five health districts, and rural Gbêkê in the Center with six health districts (see Fig. 4). This selection of the regions was done by the National Public Health Institute (INHP) overseeing epidemic/pandemic surveillance in Côte d’Ivoire. Ethical clearance was granted from the national ethics committee for life sciences and health (Comité National d’ Éthique des Science del la Vie et de la Santé ((CNESVS), No: 150–20/MSHP/CNESVS-km) in Côte d’Ivoire and a waiver was received from the responsible regional ethics commission in Switzerland (Ethikkomission Nordwest- und Zentralschweiz (EKNZ), No: AO_2020-00031). Prior to each interview, the participants were given information about the study and asked for their informed consent. The acceptability of SORMAS and its potential as an epidemiological surveillance tool was captured through semi-structured interviews with COVID-19 decision makers (district and regional directors) and SORMAS users (district surveillance officers and their deputies, healthcare providers from referral hospitals, surveillance officers of the two regions, and the officers of the national reference laboratory). We included all health personnel and decision makers involved in COVID-19 surveillance and management in the two pilot regions.

At baseline, participants were defined based on their position as future SORMAS users (surveillance officers, healthcare providers, laboratory personnel) or decision makers (district and regional directors). However, according to the function in a given institution, a participant could simultaneously act as a SORMAS user and a decision maker. Hence, at follow-up, we asked for their involvement in decision making and active use of SORMAS. Therefore, some participants answered questions on both the use of the software and decision making.

The following SORMAS user roles were assigned to the health personnel and decision makers of the two pilot regions in Côte d’Ivoire: A) at district level: surveillance officer and contact officer (two roles, same person, namely the district disease control officer), B) at regional level: surveillance supervisor and contact supervisor (two roles same person, namely the state epidemiologist). These users had full right to enter and edit all data (case and contact data, laboratory data, epidemiological history, classification of cases), but they only have access to handle data on their respective area (region or district). For the laboratory user, the laboratory officer role was given, this role has full access to all cases or contacts with samples sent for laboratory testing and can enter their results. Based on these results the surveillance officers and supervisors can re-classify or confirm the cases. The national users (INHP staff) oversees the entire data from the two pilot district, for monitoring and analysis purposes. Each user has a dashboard in SORMAS displaying the data of their respective area.

### Data collection

The questionnaire covered personal information, general knowledge on COVID-19, information on conventional surveillance systems for disease monitoring (including COVID-19), acceptability of SORMAS, and impact of SORMAS on epidemic preparedness and surveillance. The framework of Sekhon et al. with its seven dimensions of acceptability was considered: (i) affective attitude; (ii) experienced intervention burden; (iii) ethicality; (iv) intervention coherence; (v) opportunity costs; (vi) perceived effectiveness; and (vii) self-efficacy [13]. We covered these seven components in a semi-structured questionnaire, tailored to the country with the input of Ivorian stakeholders involved in the health system and disease surveillance. The adaptations and additions made to the framework were mainly related to the knowledge on COVID-19 and conventional surveillance systems, such as the COVID-19 definition and case management, as well as the country-specific control measures and conventional surveillance. For most questions on acceptability we used a five-point Likert scale, with scores of “1” being the least acceptable and “5” the most acceptable (e.g. 1 – “not at all”, 2 – “rather not”, 3 – “neutral”, 4 – “rather yes”, 5 – “yes, clearly”). The questionnaire in French was uploaded into the electronic data collection tool ODK (Open Data Kit).

In each pilot region, five to six experienced enumerators were selected and trained for two days on the specific data collection and ODK use. The testing, piloting and finalizing of the questionnaire was conducted during this training.

In July and August 2021, the Institut National d’Hygiène Publique (INHP) and the Helmholz Centre for Infection Research (HZI) conducted introduction and training events on SORMAS to future SORMAS users and COVID-19 decision makers in each pilot region. On the last day of these activities, we conducted the baseline questionnaires of this implementation study.

In March 2022, the same enumerators received a refresher training on how to conduct the survey and were given an introduction to the adapted follow-up questionnaire. The questionnaire was again tested prior to the second data collection. All respondents from the first round or their successor were contacted and an individual date for an interview was arranged, whenever possible. The second round of the survey took place in March 2022, around 6–8 months after the implementation of SORMAS and its regular use by the healthcare personnel and decision makers.

### Statistical analysis

All questionnaire data were imported for analysis in STATA v16.1 (Stata Corp. LLC, College Station, USA). We checked the data for plausibility and if needed contacted the participants for clarifications. Especially some categorization of functions (active user and decision maker) needed double-check in order to be in line with our definitions. Answers to open questions were translated from French to English for analysis. Descriptive data analysis of the scores was conducted and boxplot graphs were created to visualize the various aspects of acceptability and usability of SORMAS at baseline, before the implementation of the software, and at follow-up, after its regular use.

### Results

Overall, 136 questionnaires were applied, 70 at baseline (July and August 2021) and 66 at follow-up (March 2022). More details on the districts and health centers involved in the study and the number of interviews conducted are shown in Table 1. The majority were male (61.6%), and aged 35–44 years (35.6%). Active users were on average younger and more often female (majority 35–44 years, 43.1% women) compared to decision makers (majority 45–54 years, 32.8% women). Further information on their function, SORMAS training, and tablet possession is presented in Table 2. At follow-up, 88.6% of SORMAS users and 89.7% of participants involved in decision making reported to be trained by INHP and therefore also participated in the baseline survey.

**Table 1:** Pilot regions, districts, health centers, and number of interviews conducted at baseline and at follow-up

Region	District	Health Center	Interviews at baseline	Interviews at follow-up
**Abidjan 2**	Adjamé-Plateau-Attécoubé	Adjame General Hospital	6	8
		Urban Health Training (FSU) Attécoubé		
	Cocody Bingerville	FSU COM PMI Cocody	10	8
		Community Urban Health Center (CSU COM) of Angré		
	Treichville-Marcory	Marcory General Hospital	6	5
		Treichville General Hospital		
	Koumassi	Centre Medico Social St. Thérèse de l'enfant Jésus	7	5
		CSU COM Pangolin		
		Koumassi General Hospital		
	Port Bouet-Vridi	CSU COM Gonzague ville	7	6
		CSU COM Vridi 3		
**Gbêkê**	Bouaké Nord-Est	Mother and Child Health Service (PMI) Sokoura	6	5
		Urban Health Center (CSU) Belle ville		
	Bouaké Sud	CSU Air France	5	6
		FSU Koko		
	Bouaké Nord-Ouest	University Hospital Center (CHU) of Bouaké	8	8
		CSU of Diezoukouamekro		
		CSU Dares Salam		
	Sakassou	Rural Health Center (CSR) of Assandre	5	5
		Urban Health Center of Dibri Assirikro		
	Béoumi	CSU of Marabadjassa	5	5
		CSR of Fotobo		
	Botro	CSU of Languibonou	5	5
		CSU of Diabo		
**Total**			70	66

*CHU *Centre Hospitalier Universitaire (University Hospital Center)*, CSR* Centre de Santé Rural (Rural Health Center), *CSU* Centre de Santé Urbain (Urban Health Center), *CSU COM* Community Urban Health Center, *FSU* Formation Sanitaire Urbain (Urban Health Training), *PMI* Service de Protection Maternelle et Infantile (Mother and Child Health Service)

**Table 2:** General characteristics of study participants

		**Baseline (July/August 2021)**		**Follow-up (March 2022)**	
		**SORMAS users**	**Involved in decision making**	**SORMAS users**	**Involved in decision making**
**Participants (n)**		58	19	44	39
**Age group (n (%))**					
**25–34**		14 (24.1)	1 (5.3)	5 (11.4)	5 (12.8)
**35–44**		21 (36.2)	4 (21.1)	18 (40.9)	14 (35.9)
**45–54**		17 (29.3)	8 (42.1)	13 (29.6)	11 (28.2)
**55–62**		6 (10.3)	6 (31.6)	8 (18.2)	9 (23.1)
**Sex, female (n (%))**		27 (46.6)	8 (42.1)	17 (38.6)	11 (28.2)
**Function (n (%))**					
**Health facility level**	**Epidemiological surveillance focal point**	5 (8.6)	1 (5.3)	1 (2.3)	1 ( 2.6)
	**Healthcare provider**	21 (36.2)	1 (5.3)	25 (58.8)	15 (38.5)
**District level**	**Epidemiologic surveillance officer**	10 (17.2)	–	8 (18.2)	7 (18.0)
	**Deputy epidemiologic surveillance officer**	6 (10.3)	–	7 (15.9)	4 (10.3)
	**Director**	1 (1.7)	9 (47.4)	1 ( 2.3)	7 (18.0)
**Regional level**	**Epidemiologic surveillance officer**	1 (1.7)	–	–	1 (2.6)
	**Deputy epidemiologic surveillance officer**	3 (5.2)	–	–	–
**National level**	**Lab data manager (Institut Pasteur)**	3 (5.2)	–	–	1 (2.6)
** Others**^**a**^		8 (13.8)	8 (42.1)	2 (4.6)	3 (7.7)
**Trained at baseline by INHP (n (%))**		58 (100)	19 (100)	39 (88.6)	35 (89.7)
**Received a tablet from INHP (n (%))**		47 (81.6)	5 (26.7)	42 (95.5)	28 (71.8)

^a^deputy head of health department; head of the monitoring, evaluation and health information management department; deputy director of department; sanitary engineer; health action officer; data manager; deputy head of the health department; head of monitoring and evaluation; care unit supervisor

Out of the 66 follow-up respondents, 44 (66.7%) reported to actively use SORMAS for data entry, and 39 (59.0%) participants reported to use SORMAS in any way for decision making. These decisions could be of various types, such as case management (following up on positive cases, isolating positive cases, and confining potential contacts), surveillance and awareness raising (increasing surveillance, public awareness to control the spread of COVID-19, putting in place awareness-raising strategies and intensifying awareness in alert areas), vaccination (sensitizing the population to get vaccinated, increasing vaccination coverage, and testing more people), screening and testing (increasing the number of screenings in the area, routine sampling, and raising awareness in alert areas), and case management and contact tracing (following up on the patient and their contacts, and identifying at-risk contacts).

Among all respondents, 42.4% (*n* = 28) received regular reports containing data from SORMAS. Of these, 35.7% (10) received quarter-yearly reports, 35.7% (10) received it monthly, 7.1% (2) weekly, and 21.4% (6) received them several times per week. It is important to note that pre-defined and automated reports were not implemented during this pilot.

#### Knowledge on COVID-19 management

At baseline 69.0% (40) of active users and 73.7% (14) of decision makers knew the definition of a confirmed and a suspected COVID-19 case (positive PCR test), respectively. At follow-up, 65.9% (29) of active users and 59.0% (23) of decision makers knew the current definition of a case.

There was a better knowledge of, both active users and decision makers, on the duration of the quarantine period (91.3% of all study participants knew the correct duration) as compared to the isolation period of a case (76.9%, Table 3). Additionally, we found that overall, the knowledge concerning the quarantine period of a contact slightly decreased between baseline and follow-up (96.1% vs. 88.0%), whereas the knowledge concerning the isolation period of a case slightly increased (75.3% vs. 78.3%).

**Table 3:** Knowledge of active users and decision makers concerning the quarantine period of a contact and the isolation period of a case at baseline and follow-up

	**Baseline**	**Follow-up**
	Know quarantine period of a contact	
**Active users (% (n/total))**	94.8 (55/58)	86.4 (38/44)
**Decision makers (% (n/total))**	94.7 (18/19)	89.7 (35/39)
	Know isolation period of a case	
**Active users (% (n/total))**	75.9 (44/58)	79.6 (35/44)
**Decision makers (% (n/total))**	73.7 (14/19)	76.9 (30/39)

#### Conventional surveillance and management system

When asked about the channels used to communicate COVID-19 results to district surveillance officers or the person responsible for surveillance, respondents often mentioned more than one (Table 4). The most common form of communication was via email, both at baseline (41.4%) and at follow-up (53.0%). The least common communication method was paper at both time points. Between baseline and follow-up, there was an increase in persons reporting the use of an electronic platform and email as a means to communicate COVID-19 results.

**Table 4:** Tools for COVID-19 communication, surveillance and management activities (several answers possible)

	**Baseline (*n* = 70)**	**Follow-up (*n* = 66)**
Method of communicating results (n (%))		
SMS	8 (11.4%)	9 (13.6%)
Phone	23 (32.9%)	25 (37.8%)
Email	29 (41.4%)	35 (53.0%)
Electronic platform	16 (22.9%)	23 (34.8%)
Paper	5 (7.1%)	7 (10.6%)
Others	3 (4.3%)	2 (3.0%)
Does not know	10 (14.3%)	4 (6.1%)
Tools used for COVID-19 surveillance and management (n (%))		
DHIS2	8 (11.3%)	3 (4.6%)
MAGPI	28 (40.0%)	21 (31.8%)
SORMAS	na	47 (71.2%)
Excel	18 (25.7%)	11 (16.7%)
Notification forms	9 (12.9%)	2 (3.0%)
Book	3 (4.5%)	5 (7.6%)
Others	4 (6.1%)	6 (9.1%)
Does not know	11 (16.7%)	3 (4.5%)

As shown in Table 4, concerning the recording tool used for COVID-19 surveillance and management, at baseline most respondents used the data collection software by the company MAGPI, followed by Excel, notification forms, and DHIS2. Several months later, the use of these tools considerably decreased and seems to have been replaced by the use of SORMAS, which was by far the most commonly used tool at follow-up.

#### Acceptability of SORMAS

**Fig. 5:**
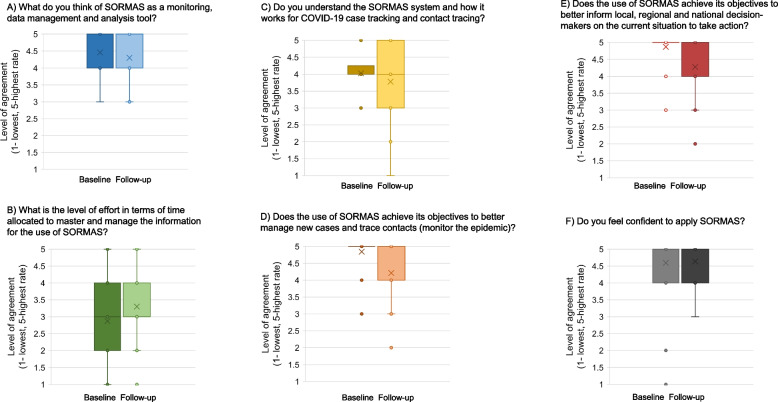
Acceptability of SORMAS at baseline and follow-up

The overall acceptability of SORMAS as a monitoring, data management and analysis tool was “positive” to “very positive” (mean 4.5 out of 5) right after the user training at baseline, and stayed on a very similar level at follow-up (mean 4.3, Fig. 5). At baseline, participants anticipated a “medium” amount of time to manage SORMAS (mean 2.9), this shifted more towards “medium to little” (mean 3.4) at follow-up. Interestingly, the answers on the understanding of SORMAS right after the training were quite uniform at good (mean 4.0, minimum 3), but after several months of using the software more or less routinely the understanding dropped slightly and was overall more diversely perceived (mean 3.7, minimum 1). Responses concerning the objectives of SORMAS “to better manage new cases and trace contacts and, therefore, monitor the epidemic and to better inform local, regional and national decision makers on the current situation to take action” very uniform and positive (Fig. 5). At baseline, respondents were clearly agreeing that SORMAS achieves its objective to better manage the epidemic (mean 4.8, 4 meaning “rather yes” and 5 “yes, clearly”). Similar results were found concerning the use of SORMAS to better inform decision makers’ action (mean 4.9). Several months after implementation, at follow-up, these two indicators decreased both to a mean of 4.2. Right after the baseline training, 94.8% (55/58) of future active users were confident (“rather yes” or “clearly yes”) to use SORMAS (mean 4.6), whereas at follow-up this decreased to 84.1% (37/58; mean 4.7). In contrast, prior to its implementation, around 14.3% (10) of interviewees expected that there would be advantages and benefits which users would have to give up due to the engagement of SORMAS, but at follow-up only 4.5% (3) of the participants raised this issue. The main concern brought up by participants was the additional time needed to enter the data in SORMAS, in addition to entering it the conventional system, MAGPI. The systematic real-time notification of COVID-19 cases meant surveillance officers had to work during additional weekends and, therefore, negatively impacted their work-life balance.

#### Ability to improve COVID-19 preparedness and surveillance

**Fig. 6:**
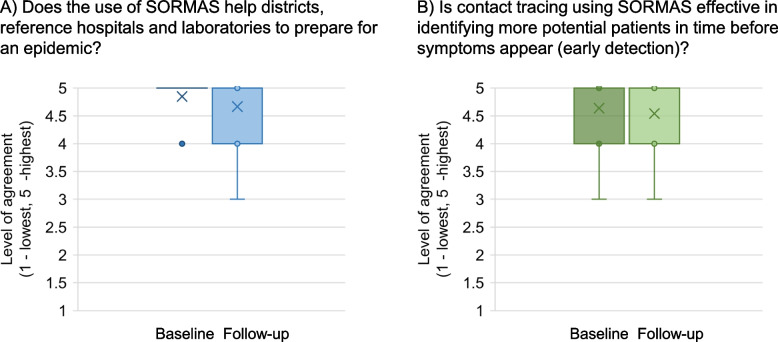
Impact of SORMAS on the level of preparedness and surveillance according to the active users at baseline and at follow-up

At baseline, active users of SORMAS were very positive that it would increase epidemic preparedness (mean 4.8, 4 meaning “yes, most cases” and 5 “yes, always”) and help early detection (mean 4.7). At follow-up these ratings decreased marginally (4.6 and 4.5, respectively; Fig. 6).

The implementation of SORMAS was reported to be smooth by most participants. Some mentioned that due to the fact that the COVID-19 case load decreased a lot during the pilot period in their health centers there was no regular use of the surveillance and management tools, which was an issue. Others experienced problems with their tablets or difficulties in synchronizing the data due to poor internet connection. Overall, many participants wished to receive a refresher training as well as to have more tablets to have the possibility to decentralize the work.

**Fig. 7:**
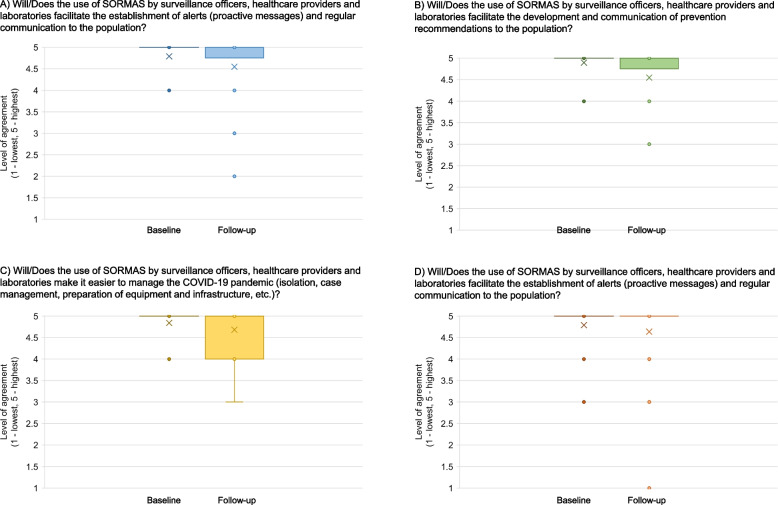
Decision makers’ expectations (baseline) and perceived experience (follow-up) on SORMAS ability to facilitate COVID-19 management and surveillance in Côte d’Ivoire

The local, regional, and national decision makers answered various questions on the ability of SORMAS to facilitate the surveillance and management of the COVID-19 pandemic (Fig. 7). At baseline, prior to the implementation of SORMAS, the expectations of decision makers were all very high. On a scale from one to five, the mean level of expectation that the use of SORMAS by surveillance officers, healthcare providers and laboratories would allow for better decision-making on risk assessment and containment measures (reports received in time with up-to-date data) was 4.8. The prospect of SORMAS to facilitate the development and communication of prevention recommendations to the population were as high as 4.9 (mean). Further, the expectation of SORMAS to facilitate the management of the COVID-19 pandemic (isolation, case management, preparation of equipment and infrastructure, etc.) reached a mean level of 4.8. The outlook on the facilitation on the establishment of alerts (proactive messages) and regular communication to the population ranked a mean of 4.8. After several months of implementation, these aspects dropped slightly but were still all between “high” and “very high” (Fig. 7). Also, at follow-up, most respondents who used SORMAS for decision-making reported that the data produced by SORMAS met their expectations to support decision-making. Their overall mean score of satisfaction was 4.5 (4 meaning “rather yes” and 5 “yes, clearly”). The four people who mentioned that the data produced by SORMAS did not meet their expectations (“rather not” (2) and “clearly not” (1)) specified further that either they never received any results emerging from SORMAS, or that it provided them with incomplete data from which they could not take decisions, or that SORMAS did not show them the total number of confirmed cases, or that they did not have the necessary tools or access to SORMAS.

During this pilot, 1585 COVID-19 cases were entered: 759 in Abidjan 2 (39 in Adjamé-Plateau-Attécoubé, 342 in Cocody Bingerville, 88 in Treichville-Marcory, 70 in Koumassi, 220 in Port Bouet-Vridi), and 826 in Gbêkê (19 in Bouaké Nord-Est, 111 in Bouaké Sud, 517 in Bouaké Nord-Ouest, 104 in Sakassou, 64 in Béoumi, 11 in Botro). After several months of more or less routine use, the main issues reported by SORMAS users were the connection that was missing between the laboratory data and the SORMAS platform, an existing feature of the software that could not be established in these two pilot areas of Côte d’Ivoire. Furthermore, SORMAS users suggested to open the use of this software for other endemic diseases that are already available in SORMAS, ensuring its synergistic and consistent routine use.

## Discussion

Côte d’Ivoire has successfully introduced SORMAS in two pilot regions (Abidjan 2 and Gbêkê) and is operating the system to surveil and manage the COVID-19 pandemic since July and August 2021. Each study region has specific characteristics; Abidjan 2 is a densely populated area and an entry point for international air and sea travel with potentially high transmission rates, whereas Gbêkê is a more rural setting with lower transmission rates. The National Public Health Institute (INHP), selected these two regions for piloting SORMAS given their characteristics and so to get an understanding how effectively and efficiently the surveillance system operates in different settings. The capital city of Abidjan also experienced much more stringent containment measures compared to the rest of the country, such as movement restrictions [14]. Overall, Côte d’Ivoire put in place a range of containment measures early in the pandemic, and then progressively reduced them over the course of the pandemic [10]. The sample size was determined by the number of health staff and decision makers involved in the surveillance, management and analysis of the COVID-19 pandemic. A limitation of the study is that a comparatively low number of health personnel and decision makers has been involved in surveillance monitoring within SORMAS, this, together with the nature of the questions, did lead to descriptive analyses rather than statistical analyses. It further limits the potential to fully extrapolate all the findings of this survey to the whole of Côte d’Ivoire, as the regions covered do not reflect the full range of different situations found across the country.

As this was a pilot study conducted during a pandemic, these two regions were simultaneously managing two other COVID-19 surveillance and management tools, namely MAGPI and DHIS2. Therefore, this pilot implementation of SORMAS and the need to enter all data on the official surveillance tools in addition to entering it in SORMAS, increased the work load of the public health staff during this exceptional time. On the other hand, it was certainly a unique opportunity to test and compare SORMAS under such conditions as the COVID-19 pandemic.

As seen in the piloting of SORMAS in Nigeria, the importance of early involvement of authorities and other stakeholders and the need for in-depth on-site training and supervision, as well as adequate technical capacity to adapt the tool to local needs are crucial [15]. This pilot profited from previous experiences of the software under non-pandemic conditions, along with SORMAS experts from HZI providing continuous technical support. On the other hand, this pilot had to work under the conditions of an ongoing pandemic which brought the health staff and infrastructure to its limits.

Containment measures and control regulations in Côte d’Ivoire were constantly adapted to the pandemic situation, based on the newest available evidence and tools. For example, the definition and handling of suspected cases changed over time, as the rapid diagnostic tests (RDT) became widely available and reliable. In the beginning of the pandemic, all suspected samples were sent to Institut Pasteur and confirmed by antigen tests using PCR. Later on, only samples providing a positive RDT were referred to PCR testing. In this study, the knowledge on the quarantine period of a contact slightly decreased between baseline and follow-up, whereas the knowledge concerning the isolation period of a case slightly increased. This may show the overall shift of focus from contact management in the beginning of the pandemic, to a more case focused management in the repression phase of the pandemic.

The self-perceived understanding of SORMAS right after the training was uniformly high, showing the usefulness and importance of the three-day long user training. After several months of more or less regular use, the self-perceived understanding of the software was rated somewhat lower and was considerably more diverse across all participants. This may be due to mobility of health workers and fluctuations in positions. For example as people who did not attend the original training, took over the task of managing SORMAS from their predecessor. Furthermore, real understanding and correct appraisal of knowledge gaps, can only be identified after regular use of a tool. Both points indicate the need for regular refresher trainings or other channels to allow the users to confidently use the software at all times. This desire for refresher trainings or other means of exchange was also directly stated by the SORMAS users in this survey. In this context, the SORMAS Foundation is developing a community platform to foster exchange through forums, groups, gamification, and file sharing (personal communication, platform: www.sormas.org).

An online surveillance system, despite being free of cost, requires equipment and its maintenance and comes with running costs for its routine operation. Therefore, the regular maintenance and exchange of tablets, as well as the host server need to be budgeted by the user country for successful and sustainable implementation of the tool. In this pilot we could see the need for additional tablets designated for SORMAS to ensure regular and decentralized data collection and entry.

SORMAS is able to connect all actors from healthcare personnel to laboratories to data managers. Unfortunately, in the pilot regions of Côte d’Ivoire the connection to the national reference laboratories was not feasible. There was already an established national notification system from the central laboratories for PCR results (trace tube). Over time, many cases were then confirmed via rapid tests, directly in the health facilities, reducing the samples being tested in laboratories. Our pilot showed that users would have liked to have the connection between the laboratories and the SORMAS database. Another process that the software would allow but was not established in Côte d’Ivoire is the delivery of regular pre-defined and automated reports. During this study, the data from SORMAS used for decision-making was depending on the individual retrieving of the data from the system. This led to some concerns of missing data or entire reports.

At baseline the largest negative impact on acceptability of SORMAS was the fear of the additional workload and time needed to handle the software. This was somehow less distinct after several months of regular use. However, there were still concerns about the additional efforts on health personnel that were already challenged during the pandemic. All districts used the national software MAGPI and to some extend DHIS2, therefore, the use of SORMAS in this pilot created not only additional work but a double entry effort. A study in Nigeria confirms that vertical programs lead to duplication of efforts, inequitable funding, and inefficiencies in surveillance [16]. The ability of SORMAS to improve COVID-19 preparedness and surveillance was rated very highly by decision makers at both time points, meaning this software would be suitable to react faster and more appropriately to a disease outbreak. Similar findings were reported in a study by Leung and colleagues that conducted an analysis of various containment strategies and found that centralized digital contact tracing tools were associated with a decline in numbers of new cases [17]. However, the national surveillance and management system MAGPI is currently not suitable for contact tracing. Opening SORMAS to other diseases was an important aspect of this pilot and was implemented during the further continuation of the pandemic. Currently, in Côte d’Ivoire, SORMAS is being used for measles, yellow fever, meningitis, cholera and COVID-19 in the two pilot regions. This ensures consistency and through the use of synergies also sustainability even after the COVID-19 pandemic has ended.

## Conclusions

The surveillance and monitoring tool, SORMAS, was successfully implemented for COVID-19, in the two pilot regions of urban Abidjan 2 and rural Gbêkê in the Côte d’Ivoire. The acceptability of SORMAS was high, but its sustainable implementation needs close supervision, regular refresher trainings and/or other channels to ensure skilled users, and maintenance of infrastructure, such as mobile devices and the host server. The potential of SORMAS for epidemic preparedness, surveillance and management with early case detection and evidence-based decision-making (containment measures, communication, management, and alerts) for infectious disease outbreaks has been shown to be substantial. By adding other endemic infectious diseases such as measles, yellow fever, meningitis, and cholera in SORMAS, it becomes a valuable tool that ensures consistency between the different diseases and uses synergies to achieve a sustainable solution. SORMAS, therefore, provides a valid, country-adapted and highly acceptable surveillance, management, and analysis tool for Côte d’Ivoire that would ensure the country to be prepared for future outbreaks and epidemics.

## Data Availability

The datasets used and/or analyzed during the current study are available from the corresponding author on reasonable request.
